# National Dental Association

**Published:** 1900-02

**Authors:** 


### Reports of Society Meetings.

### NATIONAL DENTAL ASSOCIATION.

(Continued from page 31.)

### *Third Day.—Afternoon Session.*

THE discussion of the papers read by Drs. Black and Johnson was opened, on Thursday afternoon, by Dr. Bryan (Basel, Switzerland). He said that the views expressed by Dr. Black were so opposed to the early teachings in operative dentistry as to be almost revolutionary. He was not prepared to say there was anything radically wrong in these views, but he did not feel prepared to discuss them. We need more information, more light, on these subjects. One says, the cements are the best thing we have to prevent further decay; another says, they all leak, and are porous, and are, therefore, unfit for use in the teeth; and so we seem to be all in the dark.

*Dr. S. B. Palmer* explained that his theories as to the etiology of decay were not opposed to those of Dr. Black, Dr. Miller, or Dr. Williams, but were on an altogether different plane. They represent different phases of the question. They are no more antagonistic than the links in the endless chain of evolution. From mineral to mind there is no break; all is light, order, and beauty. From mineral to vegetable, from vegetable to animal in the different stages of unfolding, a vital principle runs through the chain,—a principle which we cannot weigh, cannot measure. Dr. Palmer reviewed the steps by which he had been led to the adoption of the theory, dating from his personal experience with a retaining-plate worn in the mouth in 1847, and the peculiar effects observed in contact with food, in the generation of a distasteful current, and the experiments subsequently undertaken in the study of the subject.

*Dr. Corydon Palmer* spoke of the methods in use in earlier years, especially the use of tin, and tin and gold, in the preservation of children's teeth. Good results were obtained, and if the practice was good then, running back as far as 1839, it is good to-day, for teeth decay now just as they did then, and what preserved them then will preserve them now.

*Dr. H. H. Jackson* spoke of Dr. Black's views on the subject of environment and the constitutional causes which produce changes in the local environment; also, the effects of local deposits of matter between the teeth and in other protected localities. The teeth are hard enough to last a lifetime, if all conditions are favorable; but there are also teeth that can be cut away like chalk.

*Dr. Laurence Leonard* spoke of Dr. Black's remark that treating tooth-tissue and vigorous growth were among the prerequisites to immunity for dental caries. These conditions may be acquired by early attention to the removal of useless roots, the prompt filling of all cavities, and the proper use of the brush. These will conduce more towards good environment than the use of medicaments.

*Dr. Hofheinz* said that the cause of caries was formerly looked for in the condition of the teeth themselves, but, through Dr. Black's exposition of the subject for us, we are to abandon our reliance upon clinical experience. The patients shown by Dr. Brophy were a wonderful illustration of atavism. He wished to ask Dr. Black if, in his studies of heredity in connection with decay of the teeth, he had noted this principle of atavism.

*Dr. Black* replied that he was not prepared to answer that question without consulting his records, but he did not at the moment recall any instance of a recurrence of the conditions in the teeth of the grandparents being reproduced in the third generation, skipping the parents altogether; he was not prepared, however, to say that it was never the case.

The special order of business, a general address on "Dental Electricity," by Dr. L. E. Custer, occupied the rest of the afternoon. For Thursday night, the special order of business, the consideration of the report of the Committee on Revision of the Constitution, was followed by a continuation of the discussion on the papers of Drs. Black and Johnson. The discussion was resumed by Dr. J. N. Crouse, as follows:

*Dr. J. N. Crouse* hoped that the theory of extension for prevention, which is a debatable question, would receive free discussion.

*Dr. J. Y. Crawford* said that the papers under consideration challenge comparison with anything that has ever been written on these subjects. He said that amalgam should be eliminated from the list of materials to be used in the treatment of teeth. A low *hiss* being heard at this point, Dr. Crawford said, "That hiss will not down me when I know I am right; there is too much at stake; human life may be involved." With this exception, if Dr. Johnson's paper were printed as a leaflet, and sent to every dentist to be memorized as the Lord's Prayer is memorized by every good Christian, it would do great good.

*Dr. B. Holly Smith* said that with dentistry, as portrayed in the papers under discussion, children will no longer approach the dental chair with dread; the youngsters will come to us willingly and gladly. He said, "I congratulate the modern conservative dental operator on the new attitude of the public mind towards dentistry." It has been said that the dental profession is increasing out of proportion to the population. But with dental practice as human and conservative as advocated by Dr. Johnson, there will be fifty per cent. more done. People will no longer stay away from the dental office because of wrong impressions and dread of dental operations.

*Dr. Garrett Newkirk* spoke of the value of the combination of cement and amalgam; partly filling a cavity with cement and finishing with amalgam, thus getting the benefit of the wear of amalgam and the adhesiveness of the cement. Little children, where it is difficult to use the rubber dome, will often enjoy acting as assistant. Give them a hand-glass, adjust an absorbent pad or a piece of punk, and show them how to hold it in place between the cheek and the tooth. They will often become so interested in thus being of assistance that they will forget their own discomfort. Dr. Black having been for more than forty years engaged in this practice, and having kept complete records during that time, has facts at command; an immense mass of material from which to draw conclusions, so that the man who attempts to refute his conclusions should be sure that he is well armed.

*Dr. Darby* spoke on the subject of using arsenic in the deciduous teeth, saying that it was most generally condemned; he knows of no reason why it may not be used with proper precautions. He has used it for the past ten years, and has yet to see the first indication of unfavorable results. Of the use of gold in the teeth of young children, he said the pulp must always be taken with consideration, and the dangers from thermal changes. He considers it better practice to hold them along with cement and gutta-percha until the tooth has attained a greater degree of hardness. The results of experience and observation are entitled to more credit, and, although he did not like to pit his own observations against the results of scientific investigations, still, he could not but disagree with them as to the use of gold in young teeth.

*Dr. Bogue* regretted that more had not been said as to the value of tin and gold, and of tin alone, in the management of the deciduous teeth. He hoped the value of these materials would not be overlooked.

*Dr. McKellops* urged the use of oxyphosphate cement in children's teeth, keeping them along until it is possible to operate, as is necessary for permanent work. Even for permanent gold work a foundation of cement at the bottom of the cavity will save much time and labor. There will be no decay under it, and you can put what you choose on top of it. For old people, who cannot stand long operations, you can save the teeth for years and years with cement. If it wears away, it can be replaced with but little trouble. He said, "I am not ashamed to have any one look at my work of this character. Try it, and see what you can accomplish with it. If you will use amalgam, put cement under it, and keep the mercury away from the dentine. It will create no soreness, and it will not leak."

*Dr. Harlen* expressed his surprise at the conclusions drawn as to the penetrability of cement from cylinders placed in bottles of dye. Dr. McKellops, an old and experienced practitioner, and Dr. Darby, a professor of operative dentistry, in the face of these experiments, say that the cements will and do preserve the teeth. Laboratory experiments and practical experiments do not go hand in hand. Shall we go on using the cements, or shall we abandon them? If the experiments are to be relied upon, it is not safe to use the cements in the teeth because they leak. But it must be borne in mind that conditions in laboratory experiments are not those which obtain in the mouth. There is much, in the manipulation of the cement as mixed for use in the teeth. Some are so skilful that these cements do not either shrink or expand.

As to the use of arsenic in the deciduous teeth, Dr. Harlen said the risk was too great because of the uncertainty of retaining the application, and, if used in the molars, it may result in the non-appearance of the bicuspids, the escape of the arsenic destroying the germ of the permanent tooth. Its use for sensitive dentine has recently been reintroduced; but sooner or later it is to be apprehended that these teeth will be found to be pulpless, if not abscessed. The presence of arsenic is fatal to the vitality of the pulp. It is not safe for the majority of practitioners to use such a destructive agent. When, because of lack of physical strength or mental stamina in children, we are unable to do the thorough work we would like to do, it is better to sterilize the pulp-chamber and leave the root-canals open; even resorting to the old-fashioned plan of drilling into the pulp-chamber beneath the gum, rather than attempt ineffectual root-filling.

*Dr. Sitherwood* said that the method of manipulation, and also temperature, had a great deal to do with success or failure in the use of the cements. He has found the citrate of silver, used in the manner usual with nitrate of silver, superior to the latter in treating the deciduous teeth for the arrest of caries.

*Dr. Geo. Vaun* has been greatly indebted to the author of the phrase, "Extension for prevention." Studying carefully its full meaning has led him to greater care in the formation of cavities, with beneficial results. He has also been greatly benefited by the adoption of Dr. Bonwill's methods in the use of gutta-percha in approximal cavities in children's teeth, preserving not only the teeth themselves, but also the space in the jaw for the advent of the permanent teeth.

*Dr. W. V. B. Ames* wanted to correct the impression that there was any discrepancy between the results with cement in the teeth and in the laboratory. No claim has been made that every cement will shrink or leak in the mouth. The difference in the results is found in cements and in the dry condition, from lack of the water of crystallization. In the mouth, in contact with dentine, shrinkage is overcome because it is kept moist at all times. There are different degrees of porosity in the cements, and there are methods of overcoming this. Dr. Johnson presses the cement in hard with the finger. Dr. Pearsoll advocates the use of a tin matrix, pressing it hard against the cement, which gives a beautiful surface.

*Dr. Bogue.*—Those who come to us are those who need treatment at our hands. The whole do not need physic. He said, "I have seen sets of thirty-two teeth that were self-cleansing. I have seen a gentleman of fifty-three years of age who had never put a tooth-brush near his mouth, and his teeth were as nearly clean as any that I ever saw. He had only one spot that had needed a filling. Why was he immune? I do not think I know. The teeth were well worn, and in places the enamel had failed to coalesce, but the dentine was complete. Triturating was done accurately, and there were no deposits of it from food, no sordes. Look over the skulls in any museum and you will find many that were immune."

*Dr. Conrad.*—In the insane there is a peculiar wasting away of the cementum, a wearing away of the root-substance,—a condition not mentioned in our literature. He was sorry to see the remarks of amalgam workers hailed with applause, as he considers that amalgam has done more harm to the dental profession than all other causes put together. A low grade of dental practice makes a market for amalgam.

*Dr. Black,* in closing the discussion of this paper, said that he had limited the easy filling of children's teeth as closely as it should be limited. We must not destroy the confidence of the child, nor break down its courage, nor injure the nervous system. But to do our whole duty, we should fill permanently, at the earliest possible moment, and then there will not be so much to do in later life. There are many cases in which we are obliged to temporize; but we should endeavor to bring about the conditions that will permit of permanent work. Pulps do not die in consequence of gold fillings in children's teeth in as large a proportion as in the teeth of adults, and I have put in many as early as eight years of age.

As to the conditions of immunity, there is much that we do not know yet, but we should study the question and endeavor to elucidate it. It is an admitted fact that conditions of immunity confront us continually, they are to be found in every community. Let us seek out such cases and study them. It is not a subject for laboratory investigation, clinical study must precede laboratory work in this line. Take it home with you as a subject of thought, and report your findings.

Dr. R. Ottolengui read a paper entitled "Pronathism, Extraction, and Delay *versus* Expansion and Early Attention." This paper was a history of two cases from practice, both of prognathous upper jaw. In one case, the work was undertaken prior to the eruption of the permanent cuspids. The other case was first seen by Dr. Ottolengui when the patient was nearing her fifteenth birthday, at which time the temporary cuspids were still in place, while the first permanent molars had been extracted. Models taken by a previous operator at the age of ten showed a marked resemblance of this case at the same age, with the case first mentioned, the same teeth being present with the same malposition. In the case undertaken at the early age mentioned, the jaws were brought into good pose, the teeth in good occlusion, and asymmetry, both internal and external, secured without the sacrifice of any of the permanent teeth. The appliances used in both cases, the gradual progress at different stages, and the final results, were shown by large charts, without which description would be inadequate.

The special order of business at three P.M., Thursday, was the general address on "Dental Electricity," by Dr. L. E. Custer, Dayton, Ohio. Dr. Custer said that in the presence of the marvels of mechanical and electrical ingenuity embraced in the Niagara plant, the time and place were most opportune for the consideration of electrical science and its practical application in dentistry. After a brief review of the experiments of Galvani and Volta, the discoveries of Davy, the work of Oersted and Ampère, Faraday, Varley, Sièmens, and other noted electricians, Dr. Custer traced the progress of electric science in its application to art and science, medicine and surgery, and the advances in dental practice through modern electric science. The field of its useful application in medicine is narrow when compared with dentistry, where it is made available in the various forms of power, heat, light, and chemism. No energy at our command has such a wide range of action, and is at the same time so easily and so accurately regulated, as electricity. As a source of light and heat, it is unequalled for convenience and cleanliness and purity. As a source of heat, it ranges from holding a glass of water at the temperature of the blood to the intense heat required to fuse platinum. As a source of power, it is noiseless, when properly used. The electrolytic property of the current finds its application in cataphoresis,—for the treatment of sensitive dentine, for the projection of medicinal agents into the tissues, for lining root-canals, and for bleaching teeth. The X-ray has an appropriate and useful place as an unequalled means of diagnosis. These are but mere indications of the part electricity may take in the dental practice of the future.

The night session of the third day was devoted to the consideration of the report of the committee on amendments to the constitution, the changes proposed by the committee having been embodied in an *annotated copy* of the constitution, which was distributed among the members in order that they might be prepared to act understandingly. The amendments proposed by the committee were, with two exceptions, adopted by unanimous vote, after more or less discussion of the points involved.

The most important feature in the revision of the constitution is the provision for an Executive Council, to be composed of the president and secretary of the Association, and five members, to be elected annually by the Association. All matters of business, not otherwise specially provided for, go to the Executive Council without debate, the decisions of the Council being reported to the Association daily for ratification or rejection without discussion. The meetings of the Council are to be open to all members. The Executive Council will thus dispose of much of the routine business which has hitherto encroached upon the time which should be devoted to the discussion of scientific matters. Charges against members, resignations and reinstatements, reports of officers and Executive Committee, questions referred to the Council by the Executive Committee, and similar matters, will be relegated to the Executive Council.

A rearrangement and consolidation of the sections were also provided for, making six sections instead of ten.

The work of the sections and the section officers is also more clearly defined. Drs. Crouse, E. V. Black, and G. V. I. Brown were appointed a committee to more fully consider its methods of section work.

The amendments to the constitution were discussed by Drs. Stainton, Newkirk, Leonard, Ottolengui, Crouse, S. W. Foster, and Fillebrown.

(To be continued.)

